# Surgical resection for rectal cancer. Is laparoscopic surgery as successful as open approach? A systematic review with meta-analysis

**DOI:** 10.1371/journal.pone.0204887

**Published:** 2018-10-09

**Authors:** Marco Milone, Michele Manigrasso, Morena Burati, Nunzio Velotti, Francesco Milone, Giovanni Domenico De Palma

**Affiliations:** 1 Department of Clinical Medicine and Surgery, Federico II University, Naples, Italy; 2 Department of Surgery and Advanced Technologies, Federico II University, Naples, Italy; University Hospital Hamburg Eppendorf, GERMANY

## Abstract

**Background:**

Recently, it has been questioned if minimally invasive surgery for rectal cancer was surgically successful. We decided to perform a meta-analysis to determine if minimally invasive surgery is adequate to obtain a complete resection for curable rectal cancer.

**Methods:**

A systematic search pertaining to evaluation between laparoscopic and open rectal resection for rectal cancer was performed until 30th November 2016 in the electronic databases (PubMed, Web of Science, Scopus, EMBASE), using the following search terms in all possible combinations: *rectal cancer*, *laparoscopy*, *minimally invasive and open surgery*. Outcomes analyzed were number of clear Distal Resection Margins (DRM or DM), complete Circumferential Resection Margins (CRM) and complete, nearly complete and incomplete Total Mesorectal Excision (TME) and of patients who received laparoscopic or open treatment for rectal cancer.

**Results:**

12 articles were included in the final analysis. The prevalence of successful surgical resection was similar between open and laparoscopic surgery. About distance from distal margin of the specimen, clear CRM and complete TME there were no statistically significant difference between the two groups (MD = -0.090 cm, p = 0.364, 95% CI -0.283, 0.104; OR = 1.032, p = 0.821, 95% CI 0.784, 1.360; OR = 0.933, p = 0.720, 95% CI 0.638, 1.364, respectively).

The analysis of nearly complete TME showed a significant difference between the two groups (OR = 1.407, p = 0.006, 95% CI 1.103, 1.795), while the analysis of incomplete TME showed a non-significant difference (OR = 1.010, p = 0.964, 95% CI 0.664, 1.534).

**Conclusions:**

By pooling together data from 5 RCTs and 7 nRCTs, we are able to provide evidence of safety and efficacy of minimally invasive surgery. Waiting for further randomized clinical trials, our results are encouraging to introduce laparoscopic rectal resection in daily practice.

## Introduction

Treatment of curable, locally advanced (stage II-III) rectal cancer relies on surgical resection as decore feature of multimodality treatment process [1]. Surgical resection remains the most important treatment modality for rectal cancer in terms of curative resection, staging, prognosis and subsequent therapeutic decisions [2].

A call to action has been advocated because a high rate of inadequate surgical resection following rectal cancer surgery has been recently reported in the USA [3]. Furthermore it has been questioned if minimally invasive surgery for rectal cancer was surgical successful. Could complete mesorectal excision (TME), clear distal resection margin (DRM) and circumferential resection margin (CRM) be obtained by a minimally invasive approach?

Although in literature there is enough evidence to make the statement that post-operative complications were similar after laparoscopic or open surgery and recovery was better after minimally invasive surgery [4], high quality evidence about completeness of rectal cancer resection is lacking nowadays [5]. Thus, we decided to perform a meta-analysis to determine if minimally invasive surgery is adequate to obtain a complete resection for curable rectal cancer.

## Methods

A protocol for this review and meta-analysis was prospectively developed, detailing the specific objectives, the criteria for study selection, the approach to assess study quality, the outcomes, and the statistical methods.

### Search strategy

To identify all available studies, a detailed search pertaining to evaluation between laparoscopic and open rectal resection for rectal cancer was conducted according to PRISMA (Preferred Reporting Items for Systematic reviews and Meta-Analyses) guidelines [6]. A systematic search was performed in the electronic databases (PubMed, Web of Science, Scopus, EMBASE), using the following search terms in all possible combinations: *rectal cancer*, *laparoscopy*, *minimally invasive and open surgery*. The last search was performed on 15th July 2018. The search strategy was developed including only English speaking studies.

In addition, the reference lists of all retrieved articles were manually reviewed. In case of missing data, study Authors were contacted by e-mail to try to retrieve original data. Two independent Authors (MiM, NV) analyzed each article and performed the data extraction independently. In case of disagreement, a third investigator (MaM) was consulted. Discrepancies were resolved by consensus. Selection results showed a high inter-reader agreement (k = 1) and have been reported according to PRISMA flowchart (S1 Fig).

### Data extraction and quality assessment

According to the pre-specified protocol, all studies comparing laparoscopic and open procedures for the successful resection of rectal cancer were included. In details, the study had to provide a correct analysis of the specimen, according to the criteria of an adequate resection: (1) complete TME (total mesorectal excision; the mesorectal surface showed only minimum irregularities with a depth of less than 5 mm, no coning toward the distal margin, and smooth circumferential margin); (2) clear CRM (> 1 mm between the deepest extent of tumor invasion into the mesorectal fat and the inked surface on the fixed specimen); (3) a mean distal resection margin (>1 mm between the closest tumor to the cut edge of the tissue). Additionally, to be included in the analysis, the study had to specify that procedures were performed by expert surgeons according to validate surgical technique. In addition, according to the current literature [1,7], we decided to perform a subgroup analysis with studies reporting a nearly complete TME (the mesorectal envelope was intact except for defects no more than 5 mm deep, with no loss of mesorectal fat) and an incomplete TME. Given the characteristics of the included studies, the evaluation of methodological quality of each study was performed with the Newcastle-Ottawa Scale (NOS) [8] for non-randomized case-control studies and with the Cochrane Collaboration’s Tool for assessing risk of bias in RCTs [9]. The NOS scoring system encompasses three major domains (selection, comparability, exposure) and a resulting score range between 0 and 8, a higher score representing a better methodological quality. The Cochrane Collaboration’s Tool system encompasses seven major domains (random sequence generation, allocation concealment, blinding of participant and personnel, blinding of outcome assessment, incomplete outcome data, selective reporting, other bias) and a symbol for each domain, that correspond with high, low and unclear risk of bias. Results of the NOS scoring system and Cochrane Collaboration’s Tool for assessing risk of bias in RCTs are reported in S1 and S2 Tables.

Data regarding sample size, major clinical and demographic variables, data about distance to distal margin of the specimen for each procedure (expressed as means with standard deviation or standard error or p value) and/or the number of complete CRM and complete, nearly complete and incomplete TME between laparoscopic and open procedures were extracted.

### Statistical analysis and risk of bias assessment

Statistical analysis was carried out using Comprehensive Meta Analysis [Version 2.2, Biostat Inc, Englewood, NJ, USA, 2005] provided by Biostat Inc.

Differences among cases and controls were expressed as mean difference (MD) with pertinent 95% confidence intervals (95%CI) for continuous variables, and as Odds Ratio (OR) with pertinent 95%CI for dichotomous variables. If studies reported only the median, range, and size of the trial, the means and standard deviations were calculated according to Hozo et al [10].

The overall effect was tested using Z scores and significance was set at p<0.05. Statistical heterogeneity between studies was assessed with chi-square cochran’s Q test and with I2 statistic, which measures the inconsistency across study results and describes the proportion of total variation in study estimates, that is due to heterogeneity rather than sampling error. In detail, I2 values of 0% indicate no heterogeneity, 25% low, 25–50% moderate, and 50% high heterogeneity [11].

Publication bias was assessed by the Egger’s test and represented graphically by funnel plots of the standard difference in means versus the standard error. Visual inspection of funnel plot asymmetry was performed to address for possible small-study effect, and Egger’s test was used to assess publication bias, over and above any subjective evaluation. A p<0.10 was considered statistically significant [12]. In case of a significant publication bias, the Duval and Tweedie’s trim and fill method was used to allow for the estimation of an adjusted effect size [13].

In order to be as conservative as possible, the random-effect method was used for all analyses to take into account the variability among included studies.

### Meta regression analyses

We hypothesized that differences among included studies may be affected by demographic variables (mean age, female gender, BMI) and clinical data related to disease activity (neoadjuvant chemo- radio- or chemoradiotherapy, tumor localization expressed as the distance from the anal verge, AJCC stage).

To assess the possible effect of such variables in explaining different results observed across studies, we planned to perform meta-regression analyses after implementing a regression model with criteria of complete tumours’ resection (distal margin, complete CRM and complete, nearly complete and incomplete TME) as dependent variables (y) and the above-mentioned co-variates as independent variables (x). We also performed a meta-regression analyses after implementing a regression model with distal margin, complete CRM and TME as dependent variables (y) and the difference of the above-mentioned co-variates between the two group (laparoscopic and open) as independent variables (x), to exclude potential risk of patients’ allocation bias in open or laparoscopic group in each study.

This analysis was performed with Comprehensive Meta Analysis [Version 2.2, Biostat Inc, Englewood NJ, USA (2005)].

## Results

After excluding duplicate results, the search retrieved 4035 articles. Of these studies, 2517 were excluded because they were off the topic after scanning the title and/or the abstract, 646 for language, 686 because they were case reports, case series or reviews. Of 31 studies the on-line full-length version was not available and it was not possible to extract data from the on-line abstract. Other 143 studies were excluded after full- length paper evaluation for lack of data. Thus, 12 articles were included in the final analysis (S1 Fig) [1,7,14–24].

### Study characteristics

Of the included studies, 5 were high-quality RCTs and 7 were nRCTs. Major characteristics of study populations are shown in Table 1.

**Table 1:** Major characteristics of studies’ population.

Author [year]	Patients	Age	Females	BMI	ASA I	ASA II	ASA III	ASA IV	Neoadjuvant chemoradiotherapy chemoradiotherapy	Stage I	Stage II	Stage III	Stage IV
**Breukink [2004]**	50	68.5	36	-	-	-	-	-	61.55				
**Cheung [2017]**	334	-	-	-	-	-	-	-	-	-	-	-	-
**de’ Angelis [2016]**	104	70.75	43.88	24.88	-	-	-	-	61.55	0	19.2	52.9	27.9
**Ferko [2014]**	125	65.98	27.91	27.64	-	-	-	-	56.02	35.22	23.22	25.59	16.02
**Fleshman [2015]**	462	57.46	36.15	26.59	-	-	-	-	96.10	1.08	41.34	61.69	0
**Kang [2010]**	340	58.45	35.3	24.1	39.41	57.06	3.53	0	-	-	-	-	-
**Keskin [2016]**	587	57.82	61.61	26.07	23.53	47.73	19.55	9.18	63.01	-	-	-	-
**Ng [2014]**	80	61.15	42.5	22.75	-	-	-	-	-	13.75	32.5	43.75	10
**Perdawood [2017]**	200	67.53	29.5	26.09	32.5	51	16.5	0	24	-	-	-	-
**Ramji [2015]**	53	66.3	20.85	27.75	7.64	32.02	58.3	2.04	23.51	-	-	-	-
**Stevenson [2015]**	473	65	34.25	26.5	-	-	-	-	-	-	-	-	-
**Van der Pas [2013]**	997	64.47	38.62	26.23	22.17	59.88	19.26	0.60	-	33.9	27.18	35.91	0

Surgical resection for rectal cancer. Is laparoscopic surgery as successful as open approach? A systematic review with meta-analysis of randomized clinical trials.

The number of patients varied from 50 to 997, the mean age from 57.46 to 70.75 years, and the prevalence of female gender from 20.85 to 61.61%. Mean body mass index (BMI) varied from 22.75 Kg/m2 to 27.75 Kg/m2. Prevalence of patients with ASA Score I varied from 7.64 to 39.41%, with ASA Score II from 32.02 to 59.88%, with ASA Score III from 3.53 to 58.30% and with ASA Score IV from 0 to 9,18%. Prevalence of patients undergone to neoadjuvant chemotherapy varied from 2.85 to 100%, to radiotherapy varied from 3.85 to 98.48% and to neoadjuvant chemoradiotherapy from 23.51 to 96.10%. Prevalence of tumor localization varied from 13.2 to 53.55% for lower rectum (<5 cm from the anal verge), from 19.81 to 49.57% for middle rectum (5–10 cm) and from 0 to 51.95% for upper rectum (>10 cm). Prevalence of patients with AJCC stage I varied from 0 to 35.22%, with stage II from 19.20 to 41.34%, with stage III from 25.59 to 61.69% and with stage IV from 0 to 27.90%.

### Successful surgical resection

**Fig 1:**
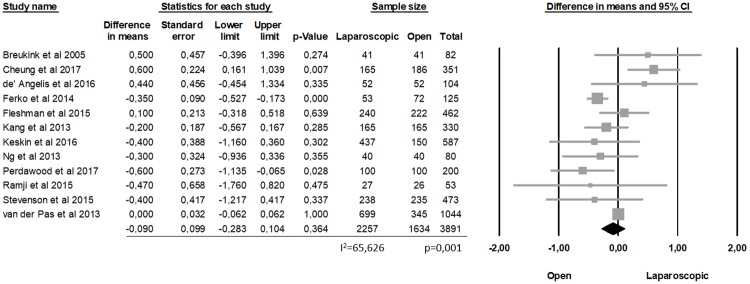
Distance from distal margin of the specimen (DRM).

The prevalence of successful surgical resection was similar between open and laparoscopic surgery. About distance from distal margin of the specimen, there was no statistically significant difference between the two groups, (MD = -0.090 cm, p = 0.364, 95% CI -0.283, 0.104), with a significant heterogeneity among the studies (I2 = 65.626%; p = 0.001) (Fig 1).

**Fig 2:**
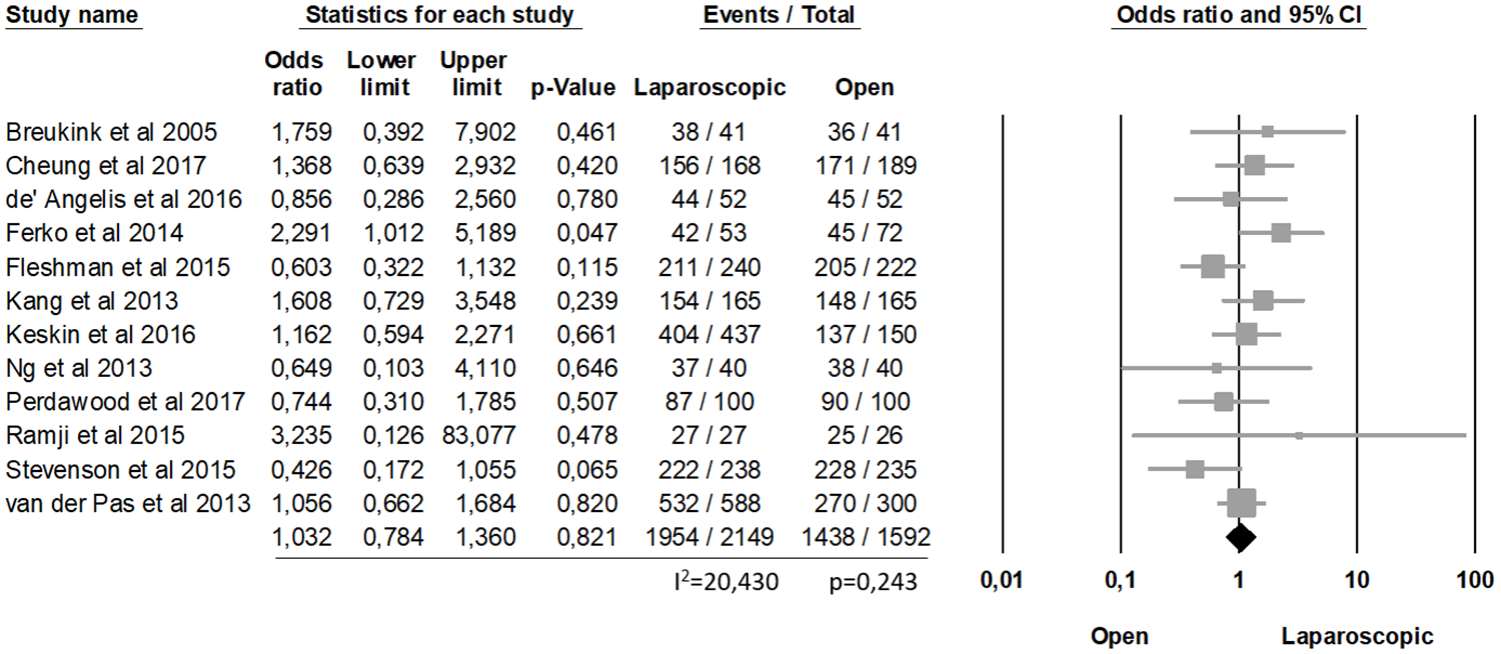
Clear CRM.

**Fig 3:**
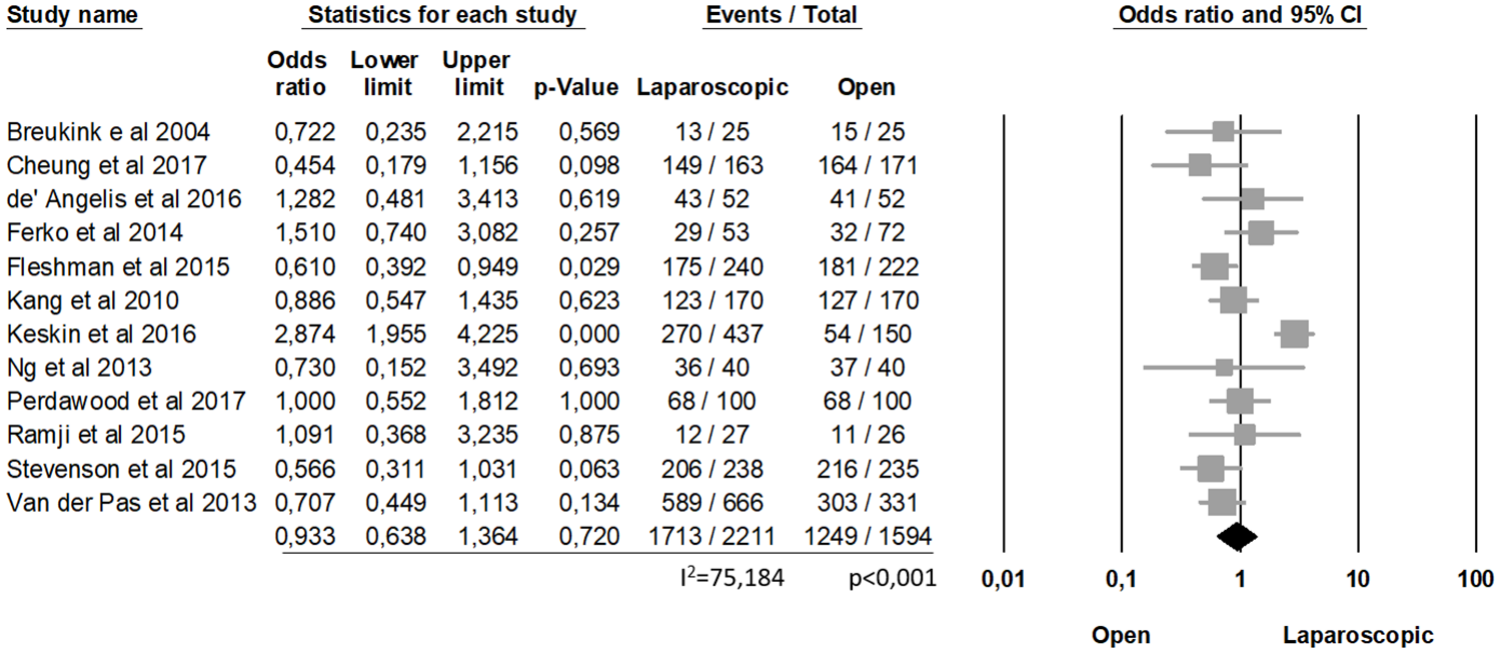
Complete TME.

The analysis of clear CRM showed no statistically differences between laparoscopic and open procedures (90.9% laparoscopic, 90.32% open, OR = 1.032, p = 0.821, 95% CI 0.784, 1.360) and no significant heterogeneity among the studies (I2 = 20.430%; p = 0.243) (Fig 2). Similarly, the complete TME was not statistically significant between the two groups (77.47% laparoscopic, 78.35% open, OR = 0.933, p = 0.720, 95% CI 0.638, 1.364), with a significant heterogeneity among the studies (I2 = 75.184%; p<0.0001) (Fig 3).

Nearly complete and incomplete TME was reported by 8 Authors [1,7,14,16–18,21,23], involving 2751 patients (1544 laparoscopic and 1207 open). The analysis of nearly complete TME showed a significant difference between the two groups (12.9% laparoscopic and 10.5% open, OR = 1.407, p = 0.006, 95% CI 1.103, 1.795), with no heterogeneity among the studies (I2 = 0.000%; p = 0.835).

**Fig 4:**
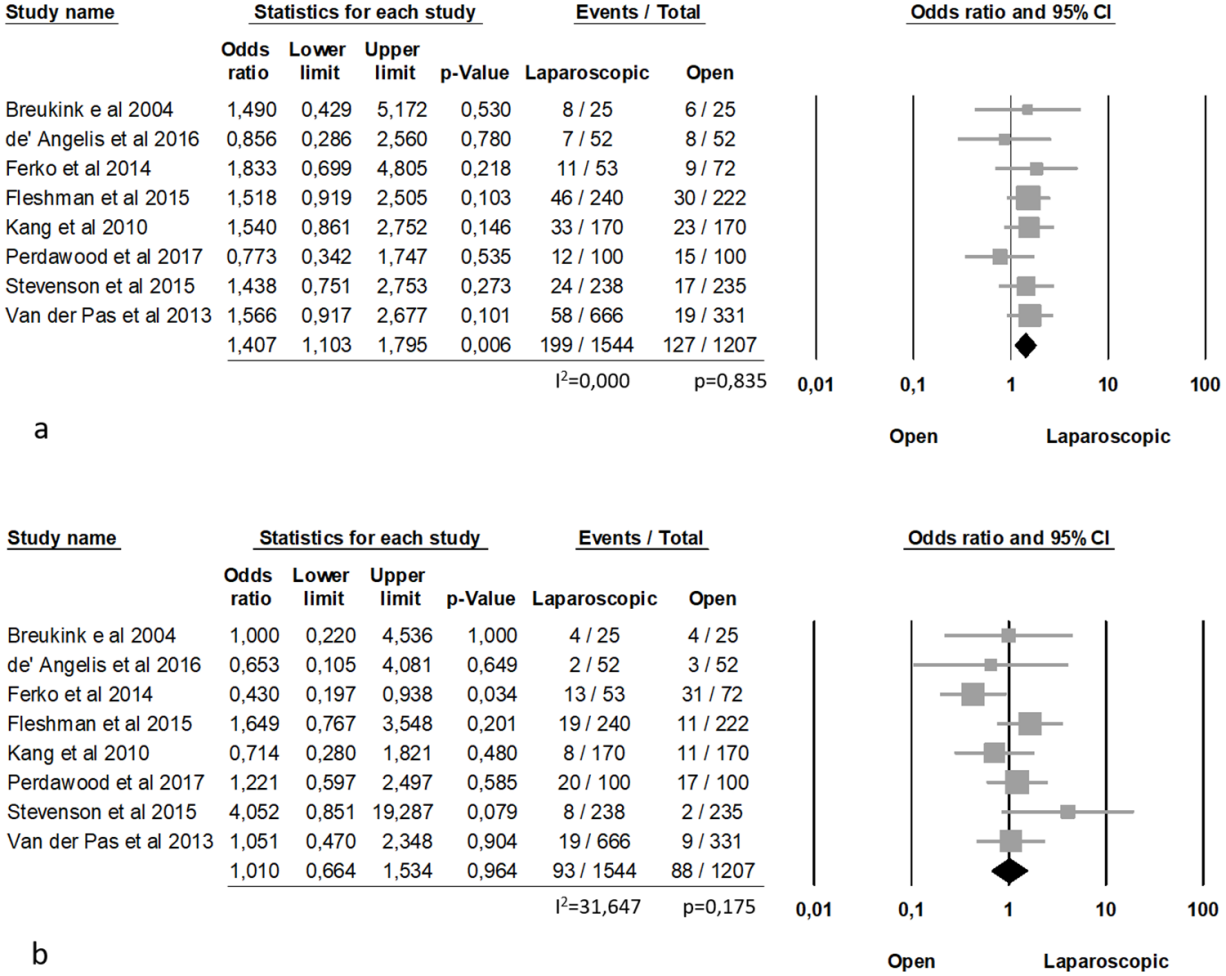
a) nearly complete TME; b) incomplete TME.

The analysis of incomplete TME showed a non-significant difference between the two groups (6% laparoscopic and 7,2% open, OR = 1.010, p = 0.964, 95% CI 0.664, 1.534) with no significant heterogeneity among the studies (I2 = 31.647%; p = 0.175) (Fig 4).

### Publication bias

Because it is recognized that publication bias can affect the results of meta-analyses, we attempted to assess this potential bias using funnel plots analysis. The distribution of studies evaluating the mean difference of the distance from distal margin of the specimen and the prevalence of complete CRM and complete, nearly complete and incomplete TME was symmetrical and no publication bias was found by the Egger’s test (p = 0.58, p = 0.75, p = 0.41, p = 0.30 and p = 0.71 respectively; S2 Fig).

### Meta-regression analysis

Regression models showed that different patients’ and tumours’ characteristics influenced various of analysed outcomes. A list of significant results is shown in S3 Table.

Complete TME was influenced by female gender, patients with ASA Score I-II, neoadjuvant chemoradiotherapy, tumour’s localization in lower, middle and upper rectum, AJCC Stage I.

Mean Distal Resection Margin was influenced by Female gender, neoadjuvant radiotherapy, tumour’s localization in lower, middle and upper rectum and by AJCC stage I and II.

Of interest, regression models showed that none of patients’ and tumors’ characteristics (age, gender, BMI, ASA Score, Neoadjuvant therapy, tumour localization, AJCC stage) influenced the number of complete CRM and nearly-complete and incomplete TME. In addition, patient allocation bias in each study could be excluded because the difference in patients’ and tumors’ characteristics between laparoscopic and open surgery did not influence the analyzed outcomes.

## Discussion

Laparoscopic approach has increasingly been performed worldwide for colorectal cancer [19].

Since its introduction in 1991, accumulating high quality evidence indicates that laparoscopic treatment of colon carcinoma is equivalent to the open technique. Moreover, high quality evidence proves that short-term and long-term safety and quality outcomes are better for laparoscopically treated patients than open patients [20, 21].

On the contrary, the role of laparoscopy in the treatment of rectal cancer is still controversial. In fact, laparoscopic rectal surgery is more difficult than colonic one, especially for middle and low rectal cancer, and the oncological safety remains unclear.

Although the proponents of the laparoscopic technique suggest that similar results in terms of oncological safety can be achieved with better short-term outcomes with minimally invasive surgery, laparoscopy in rectal cancer is still not recommended as the treatment of choice by National Comprehensive Cancer Network guidelines.

High quality data demonstrating the safety, effectiveness and oncologic appropriateness of minimally invasive resection through an abdominal approach do not exist for curable, locally advanced rectal cancer [5]. Most of the studies comparing laparoscopy to the open technique, in fact, are not randomized controlled trials and the few RCTs or meta-analysis carried-out focus on short-term outcomes [4].

Few multicentric studies have been carried out worldwide in the last 20 years. In the CLASICC trial [12] 794 patients with colorectal cancer from 27 UK centers were enrolled to receive either laparoscopic assisted or open surgery. Regarding the cancer of the rectum, no significant difference in CRM positivity was detected in patients who underwent an abdominoperineal resection in both laparoscopic and open group. CRM positivity, instead, was significantly greater in laparoscopic than in the open surgery group for patients who underwent anterior resection. As a first conclusion the impair short-term outcomes after laparoscopic-assisted anterior resection for cancer of the rectum did not justify its routine use at the time. However, it is important to underline the fact that the CLASSIC trial enrolled not only patients with rectal cancer but also patients with colon cancer, which may cause confusion on conclusions about rectal cancer. Two other multicentric trials, aimed to specifically compare laparoscopic and open surgery in patients with rectal cancer, were the COLOR II trial [23] and the COREAN trial [18], enrolling respectively 1103 and 340 patients. In the COLOR II trial a complete or nearly complete TME was obtained in 92% of laparoscopic and 94% of open procedures; CRM positivity was 10% in both groups; distal margins were negative in 100% of both procedures. In the COREAN trial TME was complete/nearly complete in 92% (laparoscopic) and 88% (open) of patients; CRM was positive in 3% of laparoscopic and in 4% of open procedures; distal margins were negative in all patients in both procedures.

In both COLOR and COREAN trials no significant differences were found regarding oncological outcomes, confirming the safety and feasibility of the laparoscopic approach for rectal cancer. On the other hand, more recently, two multicentric studies have been published, introducing controversial conclusions about the non-inferiority of laparoscopic surgery compared with open surgery. In the ALaCaRT Randomized Clinical Trial conducted between March 2010 and November 2014, 475 randomized patients with T1-T3 rectal adenocarcinoma less than 15 cm from the anal verge, underwent either laparoscopic (237) or open (238) rectal resection [7]. The primary end point was a composite of oncological factors indicating an adequate surgical resection. A successful resection was achieved in 194 patients (82%) in the laparoscopic surgery group and 208 patients (89%) in the open surgery group. The circumferential resection margins were clear in 222 patients (93%) in the laparoscopic surgery group and in 228 patients (97%) in the open group, the distal margin was clear in 236 patients (99%) in the laparoscopic surgery group and in 234 patients (99%) in the open surgery group, and total mesorectal excision was complete in 206 patients (87%) in the laparoscopic surgery group and 216 patients (92%) in the open surgery group. So, among patients with T1-T3 rectal tumors, non-inferiority of laparoscopic surgery compared with open surgery for successful resection was not established. Although the overall quality of surgery was high, the findings of this study do not provide sufficient evidence for the routine use of laparoscopic surgery, concluding that surgeons should be cautious when considering the suitability of a laparoscopic approach for a patient with rectal cancer. Similar evidence was found by Fleshman et al. in the ACOSOG Z6051 Randomized Clinical Trial [1], a multicenter randomized trial enrolling patients from 35 institutions in the United States and Canada, between October 2008 and September 2013. A total of 486 patients with clinical stage II or III rectal cancer within 12 cm of the anal verge were randomized after completion of neoadjuvant therapy to laparoscopic (240) or open (222) resection. Complete/nearly complete TME, complete CRM and DM negative was found in 95.1, 92.3 and 98% respectively for open procedures, and 92.1, 87.9 and 98% respectively for open procedures. Thus, successful resection occurred in 81.7% of laparoscopic and 86.9% of open procedures and this resulting in rejecting the non-inferiority concluding that the findings do not support the use of laparoscopic resection in patients with stage II or III rectal cancer.

In order to obtain an aggregate estimation of safety and efficacy of laparoscopic surgery for rectal cancer, meta-analyses were designed by different authors. [25–27] Particularly, in the meta-analysis by de’ Angelis et al. [28], including 14 studies, it was demonstrated that the rate of noncomplete total mesorectal excision was significantly higher in laparoscopic group, while no significant differences was found about CRM and DRM involvement when compared to open surgery group. The recent meta-analysis, by Creavin et al.[29], including the latest 4 RCTs, showed a higher complete mesorectal excision in open group, while no significant difference were showed in terms of DRM and CRM, confirming the results of de’ Angelis; in addiction, this author reported also on the grade of mesorectal excision (intact, superficial and deep mesorectal defects), underlining that it allows assessment of the quality of rectal resection and aids in the prediction of local recurrence risk. Creavin found an acceptable mesorectum (intact or superficial defects only) was present in 95,9% and 96,5% in the laparoscopic and open groups respectively, specifying that superficial mesorectal defects were more common in laparoscopic resections.

Finally, in their recent meta-analysis on 1152 operation specimens, Kitz and colleagues [30] analysed long term results of TME, founding that the TME plane was significantly associated with 3-year disease-free survival, cumulative incidence of local and distant and overall survival.

In literature there are many studies that, although they are not randomized trials, have a high scientific impact; for this reason, these studies cannot be excluded from an analysis carried out to enclose all the experience present in the literature on the laparoscopic and open approach to rectal cancer.

Thus, in order to obtain an aggregate estimation of safety and efficacy of laparoscopic surgery for rectal cancer, we realized, a meta-analysis specifically addressing the successful surgical resection and oncological radicality analyzing all relevant specimen characteristics. We focused on completeness CRM and TME and distance to distal resection margin, including all studies which provide all these three outcomes.

By pooling together data from 5 RCTs and 7 nRCTs, we are able to provide evidence of safety and efficacy of minimally invasive surgery.

In fact, there are no statistically significant differences regarding the completeness of DRM, TME and CRM, supporting the statement that laparoscopic and open approach for rectal cancer are equivalent. At difference with conclusions of de’Angelis and Creavin, our results underline that an overall analysis of literature about the completeness of rectal tumor resection, reveals a non-inferiority of laparoscopic approach despite open surgery. By this point of view, it is important to highlight that only ALaCaRT [16] and ACOSOG Z6051 [1], with their weighed impact on the meta-analysis, can determine an unbalanced influence on investigation in favour of open technique.

Moreover, in order to analyze the grade of integrity of the mesorectum, we compared all studies were completeness of TME was clearly defined as proposed by Creavin and colleagues.

Of interest, our analysis showed significant difference in the two groups in terms of nearly complete TME in favour of open approach (12.9% laparoscopic and 10.5% open) and a non-significant difference for incomplete TME. According to Creavin, we can hypothesize that superficial defects are more common in laparoscopic surgery owing to grasping or traction tears from laparoscopic instruments, ultimately resulting in discrepancies in resection specimens. It will be interesting to evaluate whether the new robotic and taTME approach, which promise even less invasiveness, will have better results.

It is worth mentioning that with our meta-regression we could assess that tumors characteristics, such as its localization, neoadjuvant therapy and AJCC stage, could be influent to the successful of resection.

The major limitation of this meta-analysis has to be addressed. All surgical resections have been performed in high volume centers by expert surgeons, leaving the doubt if these results could be extended to all surgeons worldwide. Another limitation is the influence of included cases of upper rectal cancer.

In addiction, we are waiting for further randomized clinical trials to confirm that minimally invasive surgery could be considered equivalent to open surgery in terms of oncological radicality. It is important to address that every future study cannot fail to evaluate all the histopathological characteristics.

## Supporting information

S1 Fig PRISMA flowchart of selected studies. (TIF)

S2 Fig Publication bias: a) distal margin; b) complete CRM; c) complete TME; d) nearly complete TME; e) incomplete TME. (TIF)

S1 Table Newcastle Ottawa Scale (NOS) scoring system of the included nRCTs. (DOCX)

S2 Table Cochrane Collaboration’s Tool for assessing risk of bias in RCTs. (DOCX)

S3 Table List of significant results of meta-regression analysis. (DOCX)
