# Supplementary figures and images for: Surgical resection for rectal cancer. Is laparoscopic surgery as successful as open approach? A systematic review with meta-analysis
Source: PLoS One. 2018 Oct 9;13(10):e0204887. doi: 10.1371/journal.pone.0204887 (PMC6177141; doi:10.1371/journal.pone.0204887)

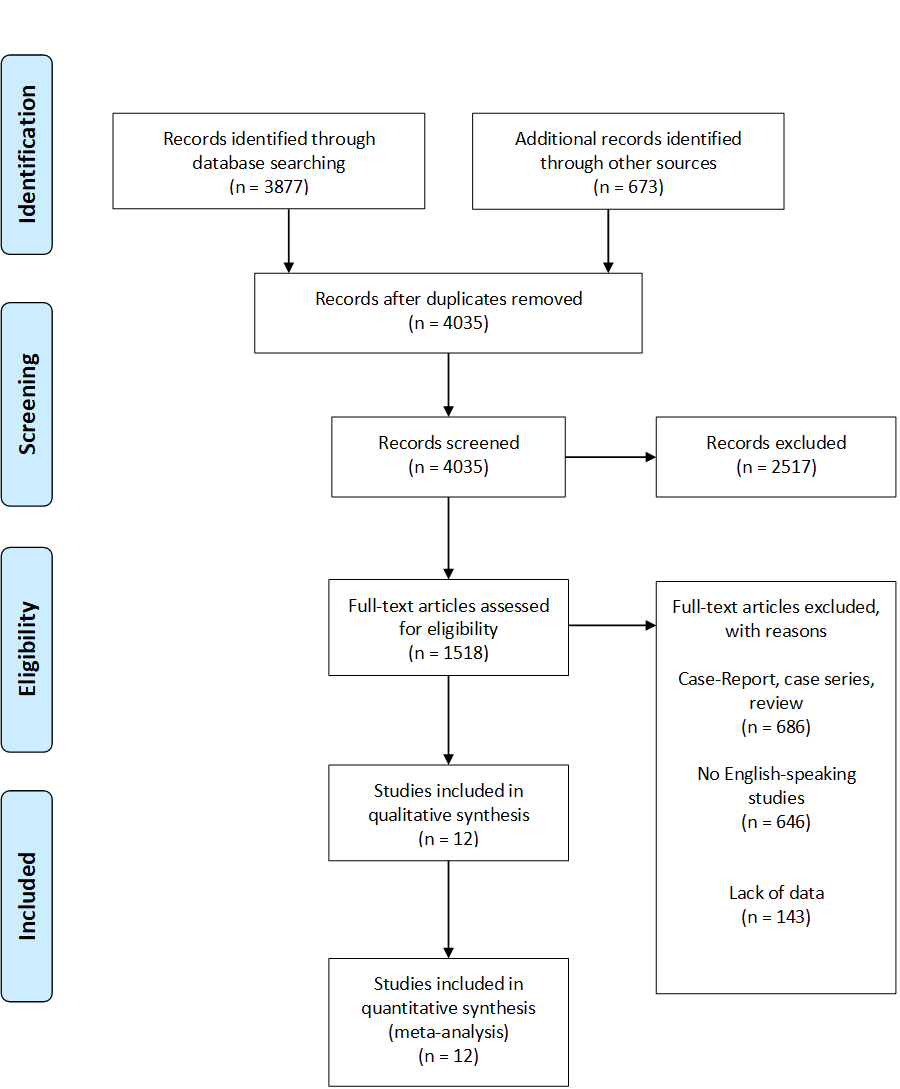

Supplement: S1 Fig — (TIF) [file pone.0204887.s001.tif]

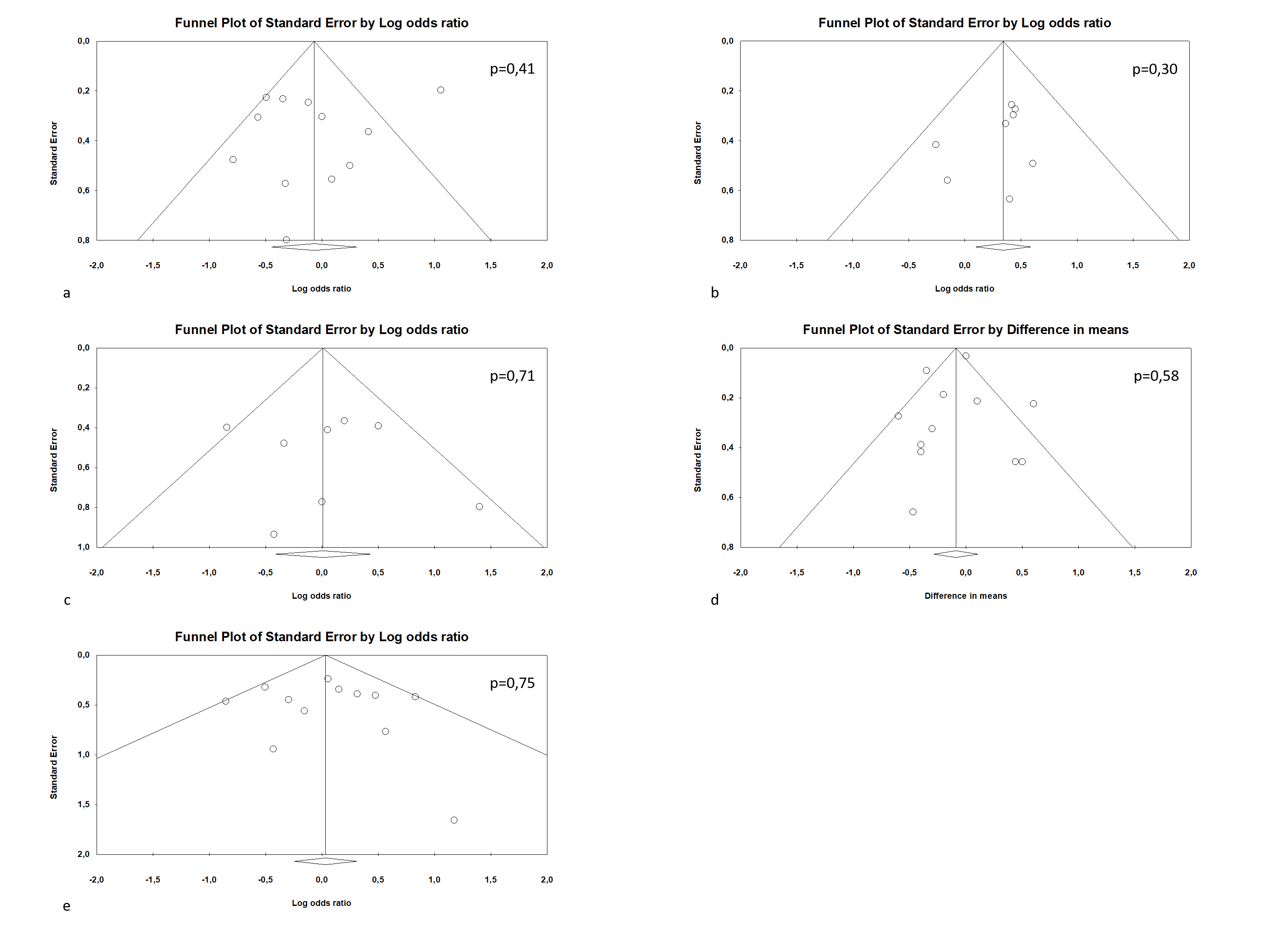

Supplement: S2 Fig — (TIF) [file pone.0204887.s002.tif]
