# Supplementary material for: Surgical resection for rectal cancer. Is laparoscopic surgery as successful as open approach? A systematic review with meta-analysis
Source: PLoS One. 2018 Oct 9;13(10):e0204887. doi: 10.1371/journal.pone.0204887 (PMC6177141; doi:10.1371/journal.pone.0204887)
Supplement: S1 Table — (DOCX) [file pone.0204887.s003.docx]

| Author [year] | Selection | |  |  | | Comparability | | |  | | Outcome assessment | | | | | |  |
| --- | --- | --- | --- | --- | --- | --- | --- | --- | --- | --- | --- | --- | --- | --- | --- | --- | --- |
|  | 1 | 2 | 3 | | 4 | | 1 | | | 1 | | | 2 | 3 | |  |  |
|  |  |  |  | |  | |  |  | | | | | | | | |  |
| Breukink [2004] | ***** |  | ***** | | ***** | | ****** |  | | | | ***** | ***** | |  | |  |
| Cheung [2017] | ***** | ***** | ***** | | ***** | | ***** |  | | | | ***** | ***** | | ***** | |  |
| de’ Angelis [2016] | ***** | ***** | ***** | | ***** | | ****** |  | | | | ***** | ***** | | ***** | |  |
| Ferko  [2014] | ***** |  | ***** | | ***** | | ***** |  | | | | ***** | ***** | |  | |  |
| Keskin [2016] | ***** |  | ***** | | ***** | | ***** |  | | | | ***** | ***** | |  | |  |
| Perdawood [2017 | ***** | ***** | ***** | | ***** | | ****** |  | | | | ***** | ***** | | ***** | |  |
| Ramji [2015] | ***** | ***** | ***** | | ***** | | ****** |  | | | | ***** | ***** | | ***** | |  |

Supporting Information 3 – Table: Newcastle Ottawa Scale (NOS) Scoring System of the included nRCTs.
