# Supplementary material for: Surgical resection for rectal cancer. Is laparoscopic surgery as successful as open approach? A systematic review with meta-analysis
Source: PLoS One. 2018 Oct 9;13(10):e0204887. doi: 10.1371/journal.pone.0204887 (PMC6177141; doi:10.1371/journal.pone.0204887)
Supplement: S2 Table — (DOCX) [file pone.0204887.s004.docx]

| **Author Random**  **[year] sequence generation** | | **Allocation concealment** | | **Blinding of participants and personnel** | **Blinding of outcome assessment** | **Incomplete outcome data** | | **Selective reporting** | | **Other bias** |
| --- | --- | --- | --- | --- | --- | --- | --- | --- | --- | --- |
| **Fleshman** +  **[2015]** | | + | | - | + | + | | + | | + |
| **Kang** +  **[2010]** | | + | | ? | + | + | | + | | + |
| **Ng [2014]** + | | + | | - | - | + | | + | | + |
| **Stevenson**  **[2015]** + | | + | | - | + | + | | + | | + |
| **[2015]** |  | | | | | | | | | |
| **van der Pas [2013]** | + | | + | - - | | + | + | | + | |

Supporting Information 4 – Table: Cochrane Collaboration’s Tool for Assessing Risk of Bias in RCTs
