# Supplementary material for: Surgical resection for rectal cancer. Is laparoscopic surgery as successful as open approach? A systematic review with meta-analysis
Source: PLoS One. 2018 Oct 9;13(10):e0204887. doi: 10.1371/journal.pone.0204887 (PMC6177141; doi:10.1371/journal.pone.0204887)
Supplement: S3 Table — (DOCX) [file pone.0204887.s005.docx]

| OUTCOMES | COVARIATES | p VALUE |
| --- | --- | --- |
| Surgical successful resection |  |  |
| Complete TME | Female gender | <0.0001 |
|  | ASA Score I | 0.04 |
|  | ASA Score II | 0.0003 |
|  | Neoadjuvant chemoradiotherapy | 0.009 |
|  | Localization in lower rectum | <0.0001 |
|  | Localization in middle rectum | <0.0001 |
|  | Localization in upper rectum | 0.0007 |
|  | AJCC stage I | 0.01 |
| Distal margin | Female gender | 0.002 |
|  | Neoadjuvant radiotherapy | 0.004 |
|  | Localization in lower rectum | 0.002 |
|  | Localization in middle rectum | 0.04 |
|  | Localization in upper rectum | 0.0002 |
|  | AJCC stage II | 0.001 |
|  | AJCC stage III | 0.0007 |

Supporting Information 5 – Table: List of significant results of meta-regression analysis.
